# Coronary Fistulas in a Patient with a Novel Long QT Syndrome Mutation

**DOI:** 10.5935/abc.20150047

**Published:** 2015-11

**Authors:** Rui Plácido, Nuno Cortez-Dias, Arminda Veiga, Cláudia Jorge, Gábriel Miltenberger-Miltény, Fausto Pinto

**Affiliations:** 1 Departamento de Cardiologia, Hospital Santa Maria University, Lisbon Academic Medical Centre, CCUL, Lisbon – Portugal; 2 Instituto de Medicina Molecular, Lisbon University Medical School, University of Lisbon, Lisbon – Portugal

**Keywords:** Long QT Syndrome, Channelopathies, Torsades de Pointes, Coronary Vessel Anomalies, Arteriovenous Fistula, Tachycardia, Ventricular

## Introduction

The congenital long QT syndrome (LQTS) is a genetic cardiac channelophathy with variable penetrance characterized by corrected QT (QTc) interval prolongation and predisposition to polymorphic ventricular tachycardia (PVT), which normally presents with syncope or sudden death. It can be caused by mutations in different ion channels, resulting in prolongation of the myocardial repolarization^1^. The clinical course of LQTS throughout a patient’s lifetime is significantly influenced by the genotype and acquired factors that additionally compromise myocardial repolarization, including drug use, serum electrolyte abnormalities, autoimmune diseases, severe bradycardia, acute heart failure decompensation and myocardial ischemia^2^.

This report presents the first description of PVT precipitated by myocardial ischemia secondary to multiple coronary fistulas in a patient with a novel LQTS-causing mutation.

## Case Report

**Figure 1:**
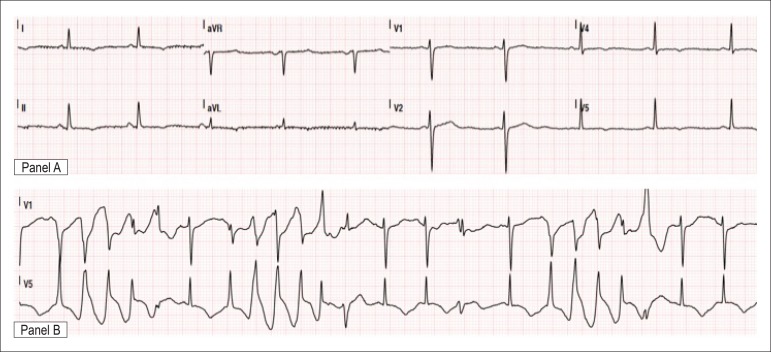
Admission electrocardiogram showing prolongation of the QTc interval (panel A) and multiples episodes of nonsustained polymorphic ventricular tachycardia (panel B).

**Figure 2:**
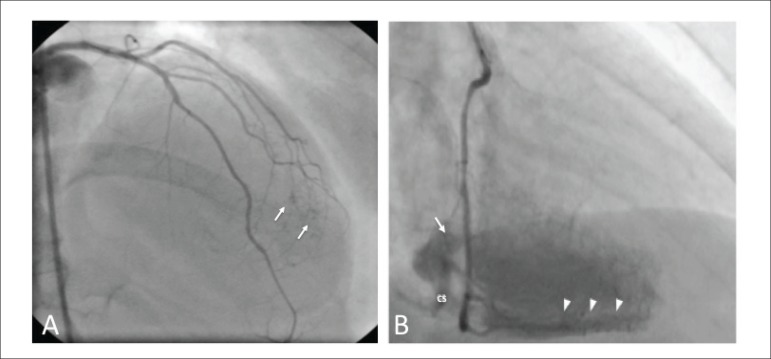
Right caudal coronariography view showing small fistulas involving the diagonal arteries (arrows).

A 47-year-old woman presented at the emergency department due to recurrent syncope within the last 2 days. Episodes occurred during moderate to extenuating exertion and were preceded by short-duration chest pain. She was a heavy smoker but no other cardiovascular risk factors were identified. During adolescence, she had been treated with phenytoin for two years for what was thought to be a seizure disorder. Since then, no further episodes of loss of consciousness had occurred and she was not receiving any medication. There was no family history of sudden death. Physical exam, chest X-ray and cranial computed tomography were unremarkable. Initial 12-lead ECG during sinus rhythm showed a marked QTc interval prolongation (640 ms) with T-wave inversion in the precordial, lateral and inferior leads (Figure 1, panel A). In addition, frequent episodes of nonsustained PVT were documented (Figure 1 – panel B). Echocardiography revealed anterior apical hypokinesia with preserved global ejection fraction. Complete blood count, serum electrolytes, renal function parameters, glycemia and thyroid hormones were normal. Troponin I was mildly elevated (0.59 ng/mL; reference range, < 0.07 ng/mL). A diagnosis of LQTS was assumed and the patient was treated with magnesium sulphate and beta-blocker, becoming asymptomatic and event-free. Coronary angiography showed no significant coronary stenosis, but fistulas from the diagonal arteries to the left ventricle cavity (Figure 2) were found. To assess the functional significance of these coronary abnormalities, the patient underwent 99mTc tetrofosmin myocardial perfusion imaging that showed at rest and under stress lower apical activity and a small area of lower radiotracer uptake in the apical segment of the anterior wall, reversible at rest. Direct sequencing of the *KCNH2, KCNQ1* and *SCN5A* genes revealed a novel heterozygous frameshift mutation in the sixth exon of the *KCNH2* gene producing a premature STOP codon: c.1232_1234delinsTTTGAA (p.Asp411Valfs*2). No pathogenic alterations were found in the other two genes. The patient was discharged with propranolol 100 mg/day, with close outpatient follow-up and plan for genetic counseling.

## Discussion

Several factors have been shown to enhance variability and prolong myocardial repolarization, predisposing to ventricular arrhythmias. In the LQTS it is possible to describe a continuous spectrum of risk for cardiac events, based on a combination of causes that prolong action potential duration and QT interval, reducing the repolarization reserve^3^. Responses to QT-prolonging factors differ among individuals. This variability in response to acquired factors is paralleled by variability in the extent to which a given mutation in the congenital LQTS prolongs QT interval and causes arrhythmias.

In the present case, we reported an unusual association between two rare cardiac conditions: the phenotypic manifestation of congenital LQTS was precipitated by myocardial ischemia secondary to coronary fistulas, based on clinical and pathophysiological criteria.

It is known that both myocardial ischemia and LQTS contribute to ventricular tachyarrhythmias that can cause sudden cardiac death. Although the arrhythmogenicity of myocardial ischemia and LQTS have been well documented, the effects of myocardial ischemia in the setting of LQTS are unclear. Several studies have suggested that both QT interval and QT dispersion, an index of the spatial inhomogeneity of the ventricular recovery times, are prolonged by ischemia^4^. Furthermore, LQTS is frequently associated with increased QT dispersion. An increased QT dispersion theoretically provides a substrate for functional reentry and has been associated with an increased incidence of malignant ventricular arrhythmias and sudden death^5,6^.

In our patient, the multiple coronary artery fistulas caused myocardial ischemia as documented by the myocardial scintigraphy. The presence of coronary fistulas is uncommon, with a reported incidence of 0.1% to 0.2% in patients undergoing coronary angiography. They can occur from any of the three major coronary arteries, but the majority arises from the right coronary or the left anterior descending arteries. Over 90% of the fistulas drain into the venous circulation. Complications include congestive heart failure due to a left-to-right cardiac shunt, myocardial ischemia, rupture of aneurysmal fistulas and endocarditis. Arrhythmias related to coronary artery fistulas are extremely rare and include ventricular tachycardia, sinus-node dysfunction and atrial fibrillation. The mechanisms are not completely understood and are probably ischemia-related^7^. The management of coronary fistulas is still controversial and recommendations are based on small retrospective series. The main indications for surgical or percutaneous closure with coils are clinical symptoms, especially of heart failure and myocardial ischemia. In our case, correction of fistulas was discouraged due to their high number and small diameter. Furthermore, beta-blocker therapy was effective in controlling ischemic symptoms and arrhythmic manifestations.

This case has another particularity, as it describes a type 2-LQTS due to a new mutation (p.Asp411Valfs*2) in the *KCNH2* (*HERG*) gene. Although this alteration is novel, pathogenicity is highly probable: the mutation leads to a frameshift and a premature termination codon two amino acids after the affected aspartic acid. We analyzed this novel alteration for pathogenicity with Mutation Taster (www.mutationtaster.org), an in-silico program that predicts the disease potential of genetic alterations. This analysis classified the p.Asp411Valfs*2 mutation as disease causing. The position p.Asp411, which is localized in the first transmembrane domain (S1) of HERG, has a special role in the protein, as it is one of the six negative charges in the voltage-sensing domain^8^. Also, p.Asp411 is creating a functional coupling with p.Lys538, at the inner end of the S4 transmembrane domain. Previous studies by Liu et al.^11^ on the function of this codon showed that in-vitro mutagenesis (p.Asp411Cys) resulting in protonation of this aspartate contributes to the signaling pathway, whereby external [H+] influences conformational changes in the channel´s cytoplasmic domains. The Asp to Val change, as seen in our patient, also leads to protonation of this aspartic acid, further supporting the pathogenicity of the novel p.Asp411Valfs*2 alteration.

LQT2 is the second most common genotype of LQTS, and occurs in 35–45% of genotyped patients with LQTS^9^. It has been shown that the clinical course of LQTS throughout an affected patient’s lifetime is influenced by genotype and genetic testing for the common subtypes of the LQTS can identify a mutation in 50 to 75% of probands in whom the diagnosis appears to be certain on clinical grounds. Risk stratification based on genetics is encouraged in some cases, probably having therapeutic implications. For example, previous observations by Moss et al.^10^ found that LQTS type 1 and LQT2 patients benefited significantly from beta-blocker therapy. Also, in a family with an affected proband and a known genetic defect, the genotyping of family members can help rule out the diagnosis.

In conclusion, to our knowledge this is the first description of the association between LQTS and multiple coronary fistulas, two rare cardiac conditions as far as we know without causal association in their primary origin. Recurrent episodes of ischemia secondary to the multiple coronary fistulas may have played an important role as precipitating factors on the genesis of PVT in this patient with a novel LQTS-causing mutation.
